# Evaluation of the Effect of Promoter Type on the Immunogenicity of the Live Recombinant *Salmonella* Vaccines Expressing *Escherichia Coli* Heat-labile Enterotoxins (LTB)

**Published:** 2018

**Authors:** Elham Mohit, Reza Nasr, Kiarash Ghazvini, Ahmad Reza Bandegi, Mohammad Reza Akbari Eidgahi

**Affiliations:** a *Department of Pharmaceutical Biotechnology, School of Pharmacy, Shahid Beheshti University of Medical Sciences, Tehran, Iran. *; b *Biotechnology Research Center, Department of Biotechnolog, Faculty of Medicine, Semnan University of Medical Sciences, Semnan, Iran. *; c *Antimicrobial Resistance Research Center, School of Medicine, Mashhad University of Medical Sciences, Mashhad, Iran. *; d *Department of Biochemistry, Faculty of Medicine, Semnan University of Medical Sciences, Semnan, Iran.*

**Keywords:** Attenuated Salmonella, ETEC, Live vaccine, LTB, Promoter, *nir*B, PhoP^c^

## Abstract

Enterotoxigenic *Escherichia coli* (ETEC)-induced diarrhoea is the second most common cause of death in children in the developing countries. Heat labile toxin (LT) is responsible for ETEC-induced diarrhoea. In the present study, a novel live ETEC vaccine based on subunit B of LT (LTB) expression in attenuated PhoP^c^ *Salmonella* strain was developed. Herein, we aimed to compare the *in-vitro* activity of promoters including constitutive *tac*, IPTG inducible* trc,* and *in-vivo*-inducible (*nir*B and *nir*B78-23) in PhoP^c^. Additionally, the ability of these recombinant PhoP^c^/pLTBs to induce LTB-specific antibody responses in BALB/c mice after nasal immunization was evaluated. *In-vitro* studies demonstrated that PhoP^c^ has the ability to produce rLTB. Furthermore, *nir*B promoter directed significantly more LTB expression in PhoP^c^/pnirBLTB under anaerobic condition without induction compared to the amount of rLTB secreted by PhoP^c^/ptrcLTB in bacterial soup under uninduced condition (6.06 ± 0.05 *vs.* 1.4 ± 0.46 μg/10^9^ cfu, *p *< 0.01). In addition, the constitutive rLTB expression from *tac *promoter was more than its expression from uninduced* trc *promoter in bacterial soup (4.2 ± 0.92 *vs.* 1.4 ± 0.46 (μg/10^9^ cfu)) and pellet (27.4 ± 0.89 *vs. *13.4 ± 1.42 (μg/10^9^ cfu), *p *< 0.0001). However, the mice immunized with PhoP^c^/ptrcLTB elicited the superior anti-LTB responses among the PhoP^c^ containing the examined prompters, which were significantly higher than those induced by PhoP^c^/pnirB78-23LTB and PhoP^c^/pnirB, 6 weeks after the first immunization. Totally, it could be concluded that *in-vitro* analysis of promoters for LTB expression in PhoP^c ^may not necessarily predict the recombinant PhoP^c ^immunogenicity.

## Introduction

The most common cause of food and water-borne *E. coli*-mediated human diarrhea is enterotoxigenic *Escherichia coli *(ETEC), worldwide. In the developing countries, the incidence of human enteric diseases caused by ETEC infections is approximately 650 million cases per year, resulting in 800,000 deaths, primarily in children of below five years old (1, 2). Therefore, an effective vaccine against ETEC for using in children living in ETEC-endemic countries and in travelers is highly required (3). 

The heat-labile enterotoxins of *E. coli* (LT) is one of the major virulence determinants for ETEC. It consists of a toxic A subunit (LTA) and a pentamer of receptor-binding B subunit (LTB) (4). The LTB bind to the gangliosides on the host cell and the A subunit enters the cell, leading to an increase in cAMP and consequently diarrhea (2). The immune-modulatory and immunogenicity properties of LTB have been extensively studied and led to its usage as a powerful candidate as immunogen and adjuvant (5, 6) in vaccine development (7).

Many vaccine candidates consisting of toxin-derived antigens either purified or expressed on the bacterial surface as live-attenuated ETEC vaccines (8, 9) have been studied and are in different stages of testing (3). Live attenuated bacteria can be engineered to deliver foreign antigens to the immune system. They are attractive vaccine vectors as they have the possibility of protection at mucosal surfaces (6, 10 and 11). The convenience to inoculate, well suited large-scale manufacturing, potential stability without refrigeration (via lyophilization) (12), strong cellular and humoral immunity induction, and also potential to be immunologic adjuvant are the other advantages of the live bacterial vaccines (10, 13). 

Growing number of reports published over the last two decades have indicated the effectiveness of avirulent *Salmonella *strains as a delivery system for recombinant heterologous antigens to stimulate protective immune response against different infectious diseases in animal models (14, 15). Several attenuations have been constructed in *Salmonella* strain to present it as avirulent while preserving different degrees of invasiveness and thus immunogenicity (16). Our group have been interested in developing a novel ETEC vaccine based on recombinant *Salmonella* strains PhoP^c^, which was attenuated for survival within macrophage (17).

Expression of a specific foreign antigen may impose considerable metabolic stress on the attenuated live vector strain, which may consequently limit its *in-vivo* immunogenicity. Furthermore, the *in-vivo* instability of foreign antigen which may be caused from unregulated and high levels of foreign protein expression is one potential drawback of using live bacteria vaccines such as *S. typhimurium *for antigen delivery. This problem can be circumvented by using* in-vivo*-inducible promoters which stabilized expression of heterologous antigens in bacterial vaccine vectors (18). The level of foreign antigen expression is low under the control of this promoter until the vector bacterium receives an environmental stimulus *in-vivo*. Consequently, they stabilized the expression of foreign antigen in live condition (18). Totally, the proper promoter to control the expression of the foreign antigen should be carefully selected for certain antigen (19). 

Herein, the effect of promoter type on the immune responses elicited by the *Salmonella*-based live vaccines expressing LTB was examined. The *in-vitro* and *in-vivo* activities of constitutive *tac* promoter, IPTG inducible *trc* promoter which have the ability to drive high-level expression of certain antigens (20) and *in-vivo*-induced promoters were compared (20). In this study the use of the *in-vivo*-induced *nir*B promoter and further derivative, which was previously constructed by our group to remove the ability of chemical induction from *nir*B promoter (21), were investigated for expression of LTB in attenuated *Salmonella* live vector vaccine. The* nir*B promoter directs the expression of an operon that includes the nitrate reductase gene. This *in-vivo*-inducible promoter is regulated by nitrate as well as nitrite and becomes active under anaerobic conditions *e.g*. inside eukaryotic cells including the macrophages (22, 23). Entry of *salmonella *into cells activated this promoter (23). *In-vivo* studies showed that the *nir*B promoter can be a highly efficient expression system for live vaccine delivery (24). Many studies demonstrated *Salmonella* strains expressing different antigens from *nir*B promoter induce higher immunity than the same strains expressing similar antigens under the control of a constitutive promoter (22, 23). 

Many studies administered *Salmonella*-vectored vaccines via nasal route in mice (25, 26) and demonstrated superior immunogenicity compared to the oral administration (27). Now, the *Salmonella*-vectored vaccines engineered to express LTB were examined in mice after nasal immunizations for stimulation of serum anti-LTB IgG antibody responses. Herein, the influence of *in-vitro* promoter activity on the induced antibody response elicited in the mice nasally vaccinated with recombinant *Salmonella *strains was evaluated.

## Experimental

*Plasmids construction*

**Figure 1:**
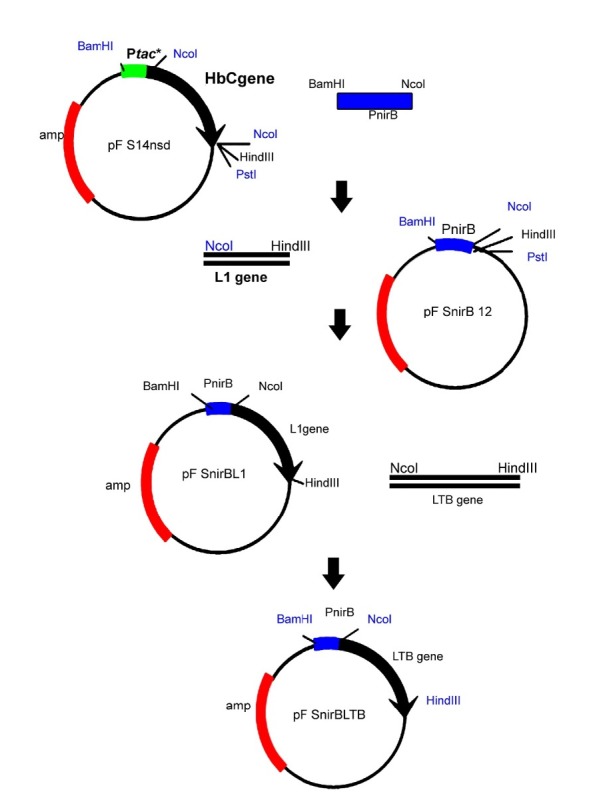
Construction of pnirBLTB. Firstly, a plasmid designated pnirB12 was constructed by cloning of the amplified *nirB Bam*HI- *Nco*I fragment in pFS14nsd, an expression vector containing HBcAg under the control of *tac*. Then, the *Nco*I-*Hin*dIII fragment encoding the HPV16-L1 open reading frame was inserted to pnirB12. Finally, the L1 fragment was replaced by the amplified LTB gene containing *Nco*I and *Hin*dIII on its end sides

To amplify *nir*B promoter, the forward primer (nirBam): (5′CGTTGGATCCAGCTGTCCGCAGGCG3′) including *Bam*HI restriction site (underlined) and reverse primer (nirNco): (5′ CTGACTGCAGCCATGGTTGCCTCGATTTC 3′) including *Nco*I restriction site (underlined) were designed based on the nitrite reductase sequence (X14202 gi:42120) and the PCR reaction was carried out using DH5α a *E. coli *genome as template. The LTB gene was ampliefied as previously reported (28). Briefly, the designed primers based on LTB gene (Gene bank accession number: J01646) were used to amlify LTB gene from a plasmid DNA of clinical isolate of enterotoxigenic containing LT operon as template. As demonstrated in Figure 1, to construct pnirBLTB, firstly the amplified *nir*B *Bam*HI-*Nco*I fragment was cloned in pFS14nsd which is an expression vector containing HBcAg under the control of *tac* promoter with a synthetic Shine-Dalgarno sequence 8 bases from the HBcAg AUG (29), a kind gift from Florian Schödel, Centre Hospitalier Universitaire Vaudois (CHUVD), Lausanne, Switzerland. The resulting plasmid designated pnirB12, was used to construct pnirBL1 by inserting the *Nco*I-*Hin*dIII fragment encoding the HPV16-L1 open reading frame (17). Then, the L1 fragment was exchanged by the amplified LTB gene (21) containing *Nco*I and *Hin*dIII on its end sides.

pnirB78-23LTB was constructed by two-step cloning as explained in our previous study (21). Briefly, the synthetic *nir*B78-23 promoter was cloned in a pkk223 derivative plasmid and then the amplified LTB gene was cloned downstream of the *nir*B78-23 promoter in *Nco*I-*Hin*dIII sites. 

For construction of ptacLTB, the LTB coding sequence flanking with *Nco*I in 5′ and *Hin*dIII in 3′ (21) was inserted in the place of L1 coding sequence in plasmid pFS14nsd HPV16-L1S (30), a kind gift from Florian Schödel, CHUVD, Lausanne, Switzerland. The construction of ptrcLTB expressing the LTB under the control of the *trc* promoter was described earlier (28). Briefly, the LT-B gene was cloned from pUCLTB into pTrc 99.

All the resulting plasmids were introduced by electroporation into the attenuated *S. typhimurium* strains PhoP^c^ (31), attenuated in both virulence and survival within macrophages by introduction of a point mutation in the *phoQ *gene (16).

*Salmonella Competent cells preparation and transformation*

To prepare *Salmonella *competent cells, the overnight cultures of different clones were diluted 1:100 and used to inoculate fresh broth LB and incubated at 37 °C with shaking to an optical density (OD)_600_ of 0.75. Next, the cells were chilled in an ice-water bath for 15 min and then were harvested by centrifugation (4,000×g, 10 min, 4 °C). Then, the cells were washed twice with ice-cold 15% glycerol. Finally, they were suspended in ice cold 15% glycerol. The plasmids were transformed into *S. typhimurium* strains PhoP^c^ by electrotransformation with a gene pulser (Biorad) set at 1.8 kV, 25 μF, and 200 Ω. After electroporation, the cells were transferred to a sterile culture tube containing SOC media (2% w/v tryptone, 0.5% w/v yeast extract, 8.56 mM NaCl, 2.5 mM KCl, 10 mM MgCl_2_ (anhydrous), 10 mM MgSO_4_ (heptahydrate) and 20 mM glucose) and incubated at 37 °C, with shaking at 150 rpm, for 1 h to allow expression of the antibiotic resistance gene. Then, the transformation mixture was centrifuged and the cells were plated on LB plates containing ampicillin (50 μg/mL) and 5-Bromo-4-chloro-3-indolyl-phosphate toluidine salt (BCIP) (50 μg/mL).

*Quantitation of expression level*

To compare the expression level of rLTB from recombinant PhoP^c^ under *nir* B78-23, *trc* and *tac* promoters, ng rLTB/10^9 ^colony-forming unit (cfu) of each strain was calculated. Accordingly, PhoP^c^ containing pnirBLTB, pnirB78-23LTB, ptrcLTB, and ptacLTB were grown in LB medium containing ampicillin (100 μg/mL) to an OD_600_ of 0.4-0.6. The PhoP^c^/pnirBLTB and PhoP^c^/pnirB78-23LTB were induced by sodium nitrite (2.5 mM), and sodium nitrate (20 mM) under both aerobic and anaerobic conditions as described previously (21). In addition, a reaction without chemical induction was also carried out. In case of PhoP^c^/ptrcLTB, and PhoP^c^/ptacLTB, the induction was performed by adding IPTG (0.4 mM). All of the recombinant PhoP^c^s was incubated for 4 h after induction. Then, cfu of each sample culture was determined. Briefly, the serial dilutions of the samples were made and 0.1 mL of each bacterial concentration was plated on ampicillin containing LB agar plate. After overnight incubation at 37 °C, the numbers of colonies on the plate were counted and then the cfu/mL *vs.* OD_600_ were plotted.

In the next step, the cells were harvested and then lysed by freezing and thawing three times with vigorous shaking. Afterwards, rLTB was measured in the crude lysates and supernatant using GM1-ELISA. The recombinant LTB concentration was calculated as ng/10^9^ cfu.

*GM1-ELISA to evaluate rLTB expression*

The amount of rLTB levels was measured by the GM1-ELISA method as describe previously (21). Briefly, polystyrene 96-well microtiter plate was coated with 5 μg ganglioside GM1 type III (Sigma) per well diluted in carbonate buffer (pH 9.6) and incubated 6 h at room temperature. Further, the plate was incubated with PBS-BSA (0.1% (w/v) (Bovine serum albumin (BSA) in phosphate-buffered saline (PBS)) for 30 min at 37 °C. Then, after washing the wells with PBS-T (0.05% (v/v) Tween-20 in PBS), 100 μL of PBS-BSA containing serially diluted standard LT (10 μg/mL) (Sigma) as positive control, diluted supernatant, and bacterial lysate of samples (1:10 to 1:10000 in PBS) were added and incubated for 1 h at 37 °C. After another washing procedure with PBS-T, the wells were incubated with 100 μL anti-LTB/cholera toxin B subunit cross reactive monoclonal antibody D15-8 (kindly provided by the Pasteur Institute, Paris) and LT39 monoclonal antibody (kindly provided by Svennerholm, university of Goteborg, Sweden) (1:10000) as the first antibody for 1 h at 37 °C. After a final washing procedure, 100 μL of tetramethyl benzidine (TMB) (Abcam) and H_2_O_2_ (at a final concentration of 0.03%) were added as the substrate, followed by incubation for 15 min at 37 °C. Then, the reaction was interrupted with 2N H_2_SO_4_. OD was measured with a spectrophotometer (Awareness Technology, Inc.) at a wavelength of 450 nm.

To evaluate the amount of rLTB expressed using different promoters in PhoP^c^, a standard curve of reference LT was generated and the amount of rLTB in each sample was determined by interpolation on standard curves. The production rate of recombinant LTB was reported as ng/10^9^ cfu.

*Immunization and serum collection*

PhoP^c^/pnirBLTB, PhoP^c^/ptrcLTB, PhoP^c^/ptacLTB, PhoP^c^/pnirB78-23LTB and PBS were used for nasal immunization. Accordingly, a single colony of each PhoP^c^ was grown at 37 °C in LB broth containing 100 μg/mL ampicillin to an OD_600_ of 0.6 to 0.8. Following centrifugation at 5,000´g for 10 min, the bacterial pellet was resuspended in 1 mL PBS to yield 0.5-5 10^11^ cfu/mL. The bacterial suspension was diluted in PBS to reach 10^9^ cfu per 20 mL.

**Figure 2:**
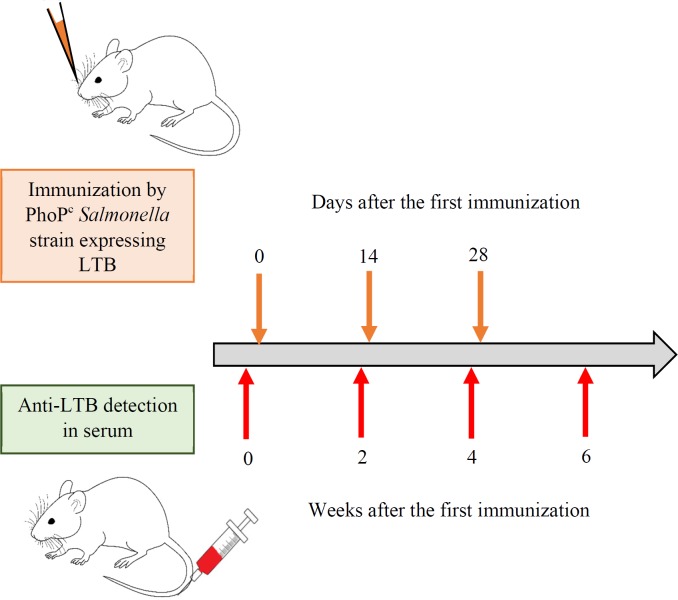
Schematic representation of immunization and anti-LTB detection. The bacterial suspension (109 cfu) of different recombinant PhoPc strains was administrated nasally after anesthetization. The booster doses were administrated two and four weeks later. The blood samples were collected form the tail vein of the mice the day before (0) of first immunization and also 2, 4 and 6 weeks after the last booster dose

Four- to eight- weeks old BALB/c mice (Semnan University of Medical Sciences) were anesthetized and 20 m L of bacterial suspension containing 10^9^ cfu of each recombinant PhoP^c^ strain was administrated by intranasal application via a Pasteur pipette. The booster doses were administrated on day 14 and 28. The blood samples were collected from the tail vein of the mice before the first immunization (0) and also 2, 4 and 6 weeks after the first dose administration (Figure 2). The serum was extracted from the whole blood by centrifugation at 4,000g for 5 min and stored at -20 °C.

*Detection of antigen-specific serum antibody responses*

The humoral immune responses to the LTB antigens were measured in the blood samples taken before (0) the first dose administration as well as 2, 4 and 6 weeks after the first immunization. ELISA plates were coated with 5 μg ganglioside GM1 and incubated at room temperature for 6 h. Then, the wells were blocked with PBS-BSA at 37 °C C for 30 min. In the next step, 100 mL of the purified LT toxin (10 μg/mL) was added to the plates and incubated for 1 h. Next, after 3 times rinsing with PBS-T, the plates were incubated for 1 h with 100 μL of serially diluted various mouse sera in PBS buffer. After a second washing step, the bound antibodies were detected using HRP conjugated goat anti-mouse antibodies at a dilution of 1:10000 in blocking solution. Immune complexes were developed with TMB in the presence of 0.03% H_2_O_2_ and the reactions were stopped after addition of 2M H_2_SO_4_.

*Statistical analysis*

Graph-Pad Prism 6.0 for Windows (Graph-Pad Prism, San Diego, California, USA) was used to perform Statistical analysis. To study *in-vitro* rLTB expression and *in-vivo* anti-LTB antibody responses, one-way ANOVA with Tukey’s post-hoc test was applied in the current study. The data were presented as mean ± standard deviation (SD). The *p*-value less than 0.05 (*p *< 0.05) was considered statistically significant.

## Results

*Expression of rLTB from PhoP* ^c^ *harboring nirB and synthetic nirB promoters*

**Figure 3:**
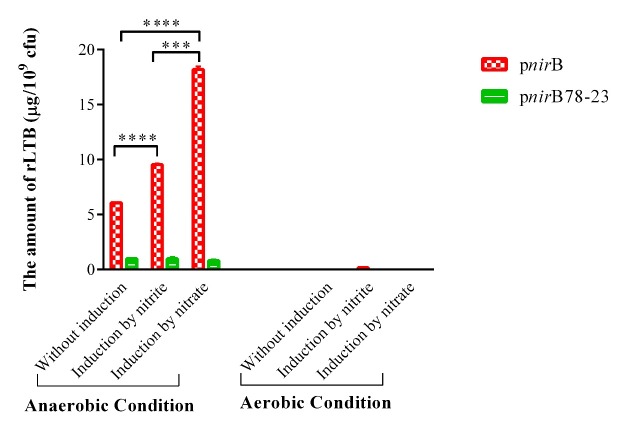
rLTB expression from PhoPc containing *nir*B and synthetic *nir*B promoters. rLTB expression was analyzed in the supernatants of PhoPc containing expression plasmids of LTB under the control of *nir*B and synthetic *nir*B promoters by the GM1-ELISA method. The data represent mean ± SD of triplicate wells. *** and **** represent for *p *< 0.001 and *p *< 0.0001, respectively

PhoP^c^ containing LTB-expressing plasmids under the control of* nir*B and synthetic *nir*B promoters were grown under a number of *in-vitro* conditions, and the supernatants were analyzed for rLTB expression by the GM1-ELISA method. No LTB was expressed in negative control, PhoP^c^ containing plasmids with *nir*B and synthetic *nir*B promoters, and without LTB gene (data were not shown). PhoP^c^ produced significant higher amount of rLTB in anaerobic compared to aerobic condition (Figure 3). As demonstrated in Figure 3, in anaerobic condition, both nitrate and nitrite significantly increased *nir*B promoter activity in PhoP^c^ *Salmonella* strain in comparison with uninduced condition (pnirB nitrate and nitrite induction *vs.* uninduced condition (*p *< 0.0001)). The *nir*B promoter activity was enhanced 1.6- and 3-fold over uninduced PhoP^c^/pnirBLTB in response to nitrite and nitrate, respectively. Significantly higher amount of rLTB was obtained in PhoP^c^ *Salmonella* by nitrate comparing to nitrite induction (pnirB, nitrate *vs.* nitrite induction, *p *< 0.0001). The efficacy of nitrate to induce *nir*B promoter in PhoP^c^ *Salmonella* under anaerobic condition was 1.9-fold of that of nitrite. However, rLTB expression under *nir*B promoter in aerobic condition was not enough to be detected by ELISA technique. Nitrite partly induced *nir*B promoter in PhoP^c^ *Salmonella* strain under aerobic condition. 

PhoP^c^ with synthetics *nir*B promoter (21) did not produce detectable amounts of rLTB when grown in aerobic condition even in the presence of nitrate or nitrite as inducers. Under anaerobic condition, the activity of synthetics *nir*B promoter was 16% of native promoter. When PhoP^c^ containing synthetics *nir*B promoter was grown under anaerobic condition, neither nitrite nor nitrate could induce rLTB expression.

*In-vitro analysis of rLTB expression by PhoP* ^c^ *uder tac and trc promoter*

**Figure 4:**
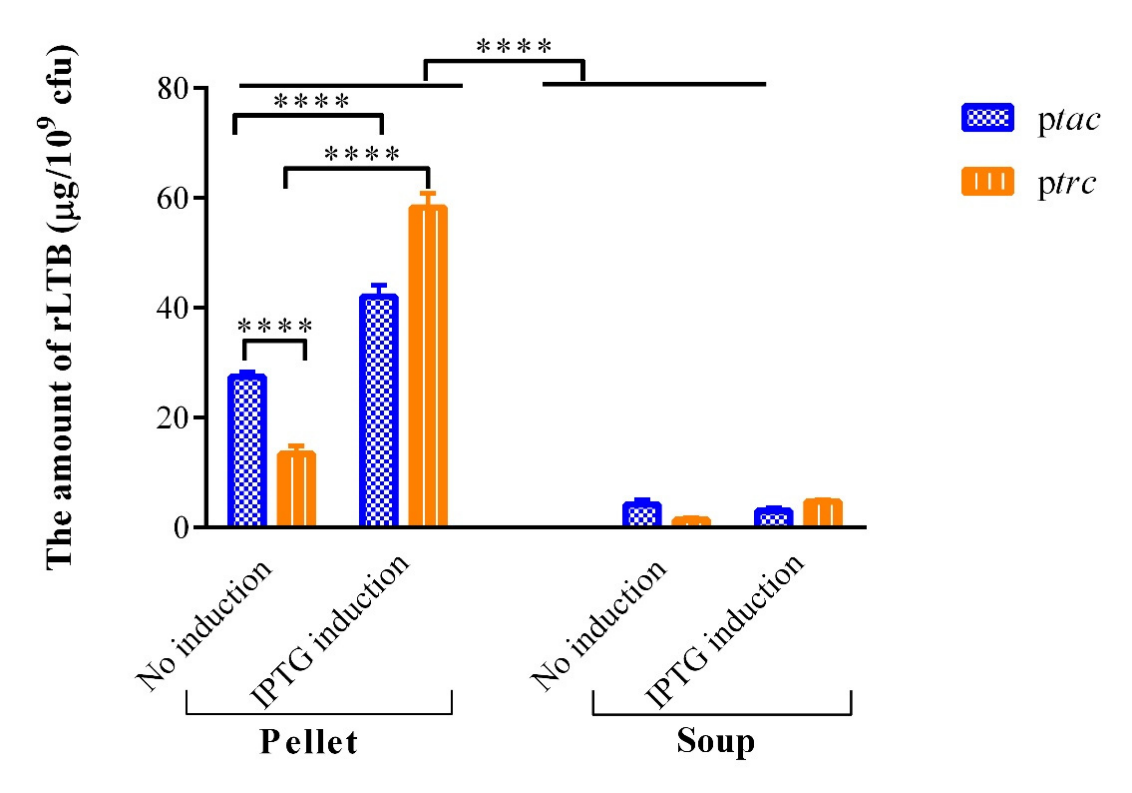
*In-vitro *analysis of rLTB expression from PhoPc under the control of *tac *and *trc *promoters. The comparison of rLTB in the lysates and supernatant of PhoPc harboring LTB-coding plasmids with *tac *and *trc *promoters was carried out by the GM1-ELISA method. Values are mean ± SD of triplicate wells. ****represents for *p *< 0.0001

The expression of recombinant LTB in the lysates and supernatant of PhoP^c^ harboring LTB-coding plasmids with *tac* and *trc* promoters was compared by the GM1-ELISA method. The rLTB was not detected in PhoP^c^ stain harboring plasmids with *tac* and *trc* promoters and without LTB gene (data were not shown). As indicated in Figure 4, the rTLB constitutive expression (without IPTG induction) under *tac* promoter was significantly lower than the induced expression in PhoP^c ^(*tac* induced (pellet) *vs.* uninduced (pellet), *p *< 0.0001). 

The results of this study demonstrated that *trc* promoter is active in both induced and uninduced conditions. IPTG induction led to significantly more rLTB expression (4.3-fold) in PhoP^c^ harboring *trc* promoter compared to uninduced condition (*trc* induced (pellet) *vs.* uninduced (pellet), *p *< 0.0001). However, the *tac* promoter in uninduced condition resulted in significantly higher rLTB expression as compared to *trc* promoter in the same condition (Figure 4, *tac* *vs*. *trc* uninduced condition, *p *< 0.0001).

Furthermore, rTLB was secreted to supernatant in both uninduced and induced conditions under *tac* and *trc* promoters in PhoP^c^. However, the amount of secreted rTLB is significantly less than in bacterial pellet in both uninduced and induced expression (pellet *vs.* soup in both induced and uninduced conditions under both *trc* and *tac* promotes *vs.* the corresponding conditions, *p *< 0.0001). Totally, it can be concluded that PhoP^c^ expressing rLTB under *tac* and *trc *promoter has the ability to be used as live vaccine vector.

*Anti-LTB humoral responses after nasal immunization*

BALB/c mice were immunized intranasally with PhoP^c^/pnirBLTB, PhoP^c^/pnirB78-23LTB, PhoP^c^/ptacLTB, PhoP^c^/ptrcLTB, and PBS. The sera were analyzed for the presence of anti-LTB antibody by an ELISA method just before (0), 2, 4 and 6 weeks after the first immunization. No anti-LTB antibody was detected in PBS-vaccinated mice (data were not shown). In response to the first dose of vaccination, the highest antibody response was elicited by PhoP^c^/ptacLTB. However, no significant differences among various groups were demonstrated after first vaccine dose. The second vaccination dose (the first boosting dose) led to more than 3-fold increase in antibody titer of the mice vaccinated with PhoP^c^/ptacLTB. However, there were no significant differences between various groups at this time. 

**Figure 5:**
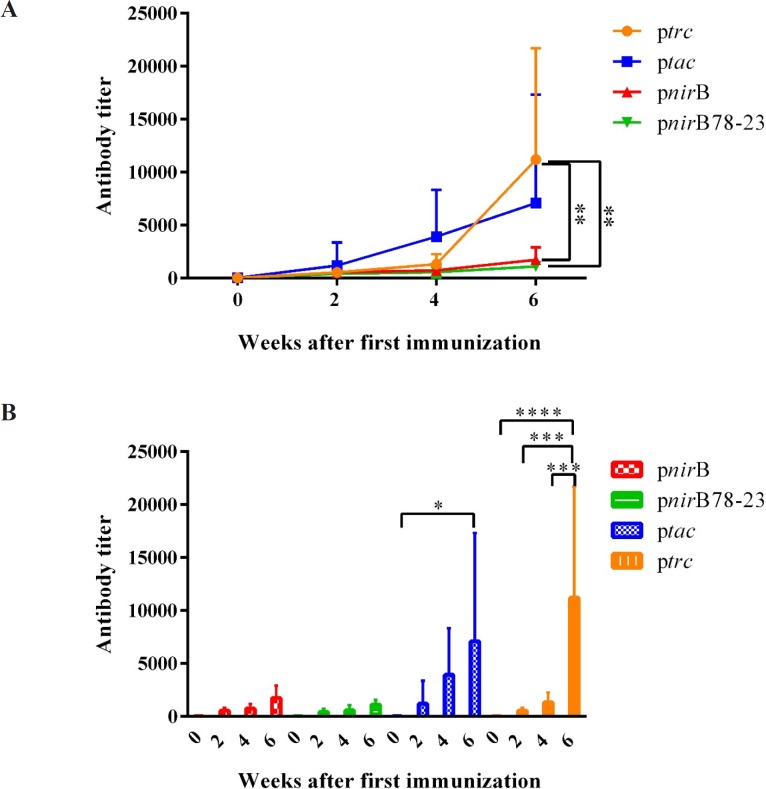
LTB specific IgG in serum after nasal immunization with PhoPc harboring different promoters. Different groups of BALB/c mice were nasally immunized with 109 cfu of PhoPc harboring *nir*B, *trc*, *tac, *and *nir*B78-23 promoters. Anti-LTB titer in the blood samples of the mice was determined the day before the first immunization (0), and also 2, 4 and 6 weeks after the first immunization. Data are expressed as the means ± SD of antibody titer of the mice in each group. (A) represents the significant differences of different groups 6 weeks after the first immunization dose. (B) demonstrates increase in antibody titer in each group after booster doses. *, **, *** and **** represent for *p *< 0.05, *p *< 0.01, *p *< 0.001, and *p *< 0.0001, respectively

The second boosting dose caused approximatey 20-fold increase in Anti-LTB antibodies in the mice received PhoP^c^/ptrcLTB. The serum anti-LTB titers induced by the second boosting dose were increased 6-, 3.2- and 2.6-fold in the mice immunized by PhoP^c^/ptacLTB, PhoP^c^/pnirBLTB and PhoP^c^/pnirB78-23LTB, respectively. As demonstrated in Figure 5A, the mice receiving PhoP^c^ strains expressing LTB under the control of the *trc *promoter demonstrated significantly higher anti-LTB antibody compared to the mice receiving PhoP^c^ strains expressing LTB under the control of the *nir*B78-23 and *nir*B promoters, 2 weeks after the second boosting dose (*p < *0.01). Totally, the data obtained in the mice using nasal immunization suggested that the PhoP^c^ strain expressing LTB from the constitutively active *trc* promoter is the superior immunogenic one.

Although PhoP^c^/pnirBLTB and PhoP^c^/pnirB78-23LTB were able to induce anti-LTB specific antibody by single dose, there was no significant increase in antibody titer even after the second boosting dose. As demonstrated in Figure 5B, the second boosting dose in PhoP^c^/ptacLTB caused significant enhancement in serum anti-LTB titer (*tac* 0 *vs.* 6 weeks after the first immunization, *p *< 0.05). In addition, each boosting dose of PhoP^c^/ptrcLTB led to a significant increase in anti-LTB titers of the vaccinated mice (*trc* 0 *vs.* 6 weeks after the first immunization, *p *< 0.0001; 2 *vs.* 4 and 4 *vs.* 6 weeks after the first immunization, *p *< 0. 001).

## Discussion

In the present study, the development of a novel ETEC vaccine based on recombinant *Salmonella* strains expressing LTB was reported. Herein, the strong constitutive *tac* and IPTG-inducible* trc* promoters as well as the *in-vivo*-inducible *nir*B and synthetic *nir*B were evaluated in *Salmonella* to quantify *in-vitro* antigen levels and to simultaneously monitor *in-vivo* antibody response. In previous studies, a range of different antigens have been expressed under the control of tac, *trc* and *nir*B promoters, in *Salmonella *strain and some of them have induced protective immunity (17, 32-35). 

Herein, *in-vitro* studies demonstrated that PhoP^c^ can produce rLTB with critical characteristics such as pentameric formation, binding to its receptor (ganglioside GM1), and conservation of immunogenic epitops under the control of both constitutive and *in-vivo* inducible promoters. In addition, the uninduced expression of rLTB in PhoP^c^ strain under *tac* and *trc* promoter was confirmed. High basal level of transcription and the leakage of *trc* promoter which cause protein expression in uninduced cultures have been proved in previous studies (36, 37). 

We previously constructed a synthetic *nir*B promoter in which the responding regions for nitrite and nitrate inducers were not considered. Consequently, the expression of recombinant protein is induced only under low oxygen pressure or anaerobic conditions (21). The synthetic promoter demonstrated less activity than native *nir*B promoter in anaerobic condition in PhoP^c ^*Salmonella* strain. In agreement with our previous report in *E. coli* (21), the synthetic *nir*B promoter did not respond to chemical inducers (nitrite and nitrate) of intact native *nirB *promoter in PhoP^c^ *Salmonella* strain. It was previously reported that the nature of the protein expressed under *nir*B and synthetic *nir*B may adversely affect due to anaerobic growth of bacteria (21). Herein, the pentameric formation of the expressed LTB which is necessary for binding to its receptor, immunogenicity and adjuvanticity was confirmed by interaction with LT39 antibody that is specific for the pentameric structure of LTB not for the monomer form (38). In addition, Guidry *et al.* demonstrated that the toxicity, immunogenicity, and oral adjuvanticity of LT are dependent on binding of the B subunit to ganglioside GM1 (39). Therefore, the results of GM1-ELISA demonstrated that receptor recognition and immunogenic determinants of the expressed LTB directed by different promoters are conserved. 

Nasopharynx-associated lymphoid tissue (NALT) appears to play a key role in induction of immune response in rodents. NALT involves M, antigen presenting cells (APCs), T and B cells and consequently provides the requirements for a protective immune response induction (25). Many studies reported higher antibody titers via the intranasal route compared to oral inoculation of mucosal vaccine (10, 25, 40 and 41). Several studies administered *Salmonella*-vectored vaccines via nasal route in mice (26, 42 and 43). Galen *et al.* demonstrated superior immunogenicity of intranasal* Salmonella typhi* live vector rather than its oral administration. They explained that the poor performance of orogastric immunization is due to the high exposure of gut to bacteria, other micro­organisms and food antigens that may suppress immune responses and limit the magnitude and duration of the induced immunity (27). Therefore, after successful *in-vitro* expressing of LTB in *Salmonella* *typhimurium* PhoP^c^ vaccine strain under the control of different promoters, we aimed to determine whether the expressed vaccine antigen could elicit humoral immune response in nasally vaccinated animals.

In the present study, we demonstrated that significant higher anti-LTB antibody titer in response to second boosting dose was achieved when the mice were vaccinated by PhoP^c^/ptrcLTB comparing to PhoP^c^/pnirB78-23LTB and PhoP^c^/pnirBLTB vaccinated mice*. *In agreement with our results, Dunstan *et al.* demonstrated that the native* nir*B promoter was not able to direct expression of immunogenic levels of C fragment in Δ*aroAD *mutant of *Salmonella*. In order to circumvent the inability of the *nir*B promoter for directing expression of immunogenic levels of C fragment, they incorporated optimal RBS sequence containing complementary sequence of *E. coli* 16S rRNA and also optimal size of the spacer region between the RBS and ATG, into the *nir*B promoter construct. They demonstrated that the incorporation of the optimal RBS had no effect on the anti-tetanus toxoid antibody generated in the mice immunized with *S. typhimurium *strains expressing the C fragment of tetanus toxin using *nir*B promoter. Therefore, they concluded that the poor translation initiation is not responsible for the lack of immune response from the *nir*B promoter cassette (44). However, some studies reported that *Salmonella* strains expressing antigens under *nir*B promoter demonstrate superior immunogenicity than the similar strains in which the expression of the same antigens is controlled by a constitutive promoter (23, 35). For example, our results are in contrast with the study of McSorley *et al.* in which gp63 expressing under either inducible *(nir*B and *osmC*) or constitutive (*tac*) promoter in *S. typhimurium *were compared. They demonstrated that *nir*B shows superior plasmid stability which correlated with the increased immunogenicity to the heterologous antigen gp63. Furthermore, significant cellular immune responses were detected only in the mice immunized with a single dose of *S. typhimurium *expressing gp63 under *nir*B promoters. However, these responses were not detected in the mice vaccinated with a single dose of *S. typhimurium *expressing gp63 under either *tac* or *osmC* (18). 

The aim of the most studies using attenuated strains of* Salmonella* as live vaccine vectors is to determine the most important factor for the development of an immune response against a foreign antigen (45). In earlier study, it was demonstrated that the immunogenicity of the vaccine is influenced by the amount of antigen produced and consequently the choice of promoter. It was believed that more antigen production by strong constitutive promoters lead to more immunogenic vaccine (10). Cardenas *et al.* demonstrated that the initial amount of antigen that primes the GALT is more important than the persistence of the vector in tissues for eliciting immunity against a foreign antigen orally administrated by attenuated strains of *Salmonella* (46). However, some studies demonstrated that *in-vitro* study of antigen expression by live vaccine may not accurately correlate to *in-vivo* immunity. For example, John *et al.* compared the strong constitutive *tac *promoter, the *in-vivo* induced *htpG *promoter, and the *in-vivo*-induced iron-regulated *irgA *promoter for the expression of B subunit of cholera toxin (CtxB) in *V. cholera. *They demonstrated that under *in-vitro* condition, the expression was greatest and equivalent from the *tac* promoter and the *irgA* promoter when under low-iron conditions. Although the gut is a low-iron environment, *V. cholerae* strains expressing CtxB from the *tac* promoter directed superior immune responses in animals (20)*.* In another study, despite the fact that the levels of *in-vitro* expression of C-fragment of tetanus toxin under *in-vivo*-inducible *pagC* promoter and constitutive *trc* promoter in *Salmonella* appeared to be equivalent, it was found that the *pagC* promoter stimulates greater immune responses than the *trc* promoter (44). In a study by Chatfield *et al.*, *in-vitro* study indicated equivalent or higher expression levels of C-terminal of tetanus toxin (Frg C) in *Salmonella* strain under the control of *tac* promoter in comparison with the strain with the p*nir*B-Frg C construct. However, less immunity against tetanus was achieved in the mice immunized with P*tac*-Frg C *Salmonella* strain (35).

In our study, *in-vitro* studies demonstrated that more rLTB is expressed by the constitutive *tac* promoter compared to the uninduced *trc* promoter in bacterial soup (4.2 ± 0.92 *vs.* 1.4 ± 0.46 μg/10^9^ cfu( and also pellet (27.4 ± 0.89 *vs.* 13.4 ± 1.42 μg/10^9 ^cfu, *p *< 0.0001). Furthermore, *nir*B promoter directed more LTB expression in PhoP^c^/pnirBLTB under anaerobic condition without induction compared to the amount of rLTB secreted by PhoP^c^/ptrcLTB in bacterial soup (6.06 ± 0.05 *vs.* 1.4 ± 0.46 μg/10^9 ^cfu, *p *< 0.01). However PhoP^c^ strains expressing LTB under the control of the *trc *promoter stimulated superior immunity. Our results indicated that of the promoters examined, the *trc* promoter may be the best suited promoter for expression of LTB in PhoP^c^ strains. Totally, consistent with the results of Dunstan *et al.* it can be concluded that the strain’s immunogenicity poorly correlates with the total amount of heterologous antigen that a vaccine strain produces (44). Due to the complexity of living vectors interactions with mammalian hosts, *in-vitro* study is not enough to predict *in-vivo *responses (10). A number of other variables, such as the location, timing, as well as the identity of the antigen to be expressed and the type of required immune response may have influence on the efficacy of vaccine based on attenuated *Salmonella* (20, 44)*.*

In summary, anti-LTB humoral response was used as a surrogate marker to compare *in-vivo* activity of various promoters in PhoP^c^. Despite the fact that the level of rLTB expressed constitutively *in-vitro* (without IPTG) under *tac* promoter was more than that expressed under *trc* promoter, it was found that immunization using recombinant PhoP^c^ under the control of *trc* promoter results in the superior humoral immunity. Consistent with Dunstan *et al.*, we concluded that the level of *in-vitro* antigen expression is not the only factor affecting the efficacy of attenuated *Salmonella*-based vaccine (44). 
